# Medical Association of Central New York

**Published:** 1906-07

**Authors:** C. A. Greenleaf

**Affiliations:** Secretary, of Rochester; Buffalo


### SOCIETY PROCEEDINGS.

### Medical Association of Central New York.

*(Concluded from page 661, June.)*

Reported by Dr. C. A. GREENLEAF, Secretary, of Rochester.

(Edited by WILLIAM C. KRAUSS, M. D., Buffalo.)

DISCUSSION

A. B. MILLER, Syracuse: I have in mind a case where the patient was suffering with hernia. Owing to her extreme weight it was doubtful whether she would stand anesthesia. Depending upon the strength of her heart instead of lungs an attempt was made to give her chloroform. It was soon apparent that she could not stand the chloroform, and it was necessary to resort to oxygen to restore her. She was removed to the operating room and cocaine was injected about the hernia and the operation was completed in about twenty-five or thirty-five minutes under the influence of cocaine. The patient's weight was 290 pounds. The operation was done by the Mayo method. The omentum being involved, it was necessary to remove quite a large part of the omentum. The only time she complained was while a gut suture was being introduced.

JOHN O. ROE, Rochester: Having had considerable experience in the use of local anesthesia, I regard it as second only to general anesthesia in general surgery, and in minor surgery it is often decidedly preferable. By local anesthesia I am able to perform operations about the nose and throat which before were regarded impossible without excruciating pain. For instance, in the replacement of fractured noses and also in re-fracturing for correcting deformities of the nose, I am able with local anesthesia to make these operations absolutely painless. The same is true in operations for deviations of the septum, in which I never use general anesthesia, except in children. The advent of cocaine I think has done more for minor surgery than any recent discovery. It, together with adrenalin, has made operations in the nose possible that before were regarded as impossible and were not performed. The same may be said of ophthalmic surgery. There is one local anesthetic I wish to speak of particularly, which I find very valuable for subcutaneous or submucous use. It is called Wilson's Local Anesthetic. It is made by the brother-in-law of Dr. Case Jones of Rochester. Although Dr. Jones could not tell me exactly its composition, he said it was composed of cocaine, eucaine and some other preparations of a similar nature in small quantities, and also that it contains some trinitrin to counteract any depressing effects, and that it is non-toxic.

In tonsillectomy and in the removal of growths from the nose, throat and neck, the operation can be made practically, and in most cases absolutely, painless. I have found this preparation very rapid in is effects and far superior to solutions of cocaine, and even in cases where it has been necessary to use two or three drachms for one operation, I have never seen any toxic effect whatever, even in patients particularly susceptible to the effects of cocaine.

M. B. TINKER, Clifton Springs: I would like to say a word in relation to this subject, for the reason that I have used local anesthesia for over seven years in a great many hundred operations, including a great many major operations as well as minor operations of surgery, and with the greatest of satisfaction and success. Among the operations I have done personally under local anesthesia are prostatectomy, excising the glands of the neck, not only superficial, but along the jugular vein and carotid arteries. I have done many hernias. I never operate with general anesthesia unless the patient objects to the local anesthesia. I have operated on patients under fifteen and over eighty. I have used it in cholecystitis in cases of typhoid fever. I have seen it used for appendectomy, but I can not say it is as successful. I would like to mention in connection with this subject what may not be familiar to some of the members present, the use of *scofalmia*. It has been used by the Germans in the same hospital in Germany for a number of years, and only recently has it been discovered that it has a general anesthetic effect as well. Certain German operators have reported as many as two hundred operations. The *scofalmia* is injected with morphine, about one-sixteenth of a grain at first, a second dose an hour and a half before operation, and a third dose a half hour before operation. I have tried the dose in half the amount used by the Germans. I have a patient now recovering, two weeks after the operation for excision and removal of the rectum and stitching it up, a patient who has a double heart murmur. I used there *scofalmia* in connection with the local infiltration of an anesthetic. Almost any of the major operations of surgery can be performed under local anesthesia with relative or little pain if you are forced to it. In certain operations I would be strongly inclined to use the general anesthetic if I felt the patient's condition would admit of it, but if I cannot I do not give up the operation, for the reason that I have seen repeatedly, patients who have stood a difficult operation with relatively or little pain where it seemed almost an impossibility. There is much to say about the subject which I would like to say. For a person who wants to do minor operations, one should use weak solutions of cocaine. It is better to use one-half of one per cent.

of it and inject ten drachms, than it is to use one drachm in one per cent. solution. The infiltration is a great factor in the anesthesia. I mention the weak solution, because there has been a great deal written about the poisonous effect of cocaine. If you use weak solutions you can inject as much as a half pint of solution in high operations for infiltration without poisonous effects.

The President called for the report of the committee appointed to draw up suitable resolutions upon the death of Doctor Didama.

### IN MEMORIAM—HENRY DARWIN DIDAMA

Full of years and honors after long and active service in his profession, Dr. Henry Darwin Didama, one of the charter members of the Central New York Medical Association has ceased his earthly labors and gone to that long rest to which his years and labors entitle him.

From the birth of the Association until the hour Doctor Didama closed his eyes his interest in its welfare was never found wanting; so long as his physical condition allowed he was an eager worker in the ranks, ready to aid by his material and moral support.

His life was full of worthy examples and sacrifices. Doctor Didama was a leader in the profession, a man of strong personality, full of energy, at all times a student, one whose influence was far-reaching, noble and always exerted in behalf of his profession and suffering humanity.

He lived to see the profession to which he dedicated his life completely metamorphosed. It was his pride to follow the giant strides of the investigator and clinician and to teach that which was most advanced, that only which he had proved to be of value after painstaking observation.

Doctor Didama was a revolutionist in practice, proving all things for himself without hastening to conclusions which were unjustified by trial. He was a staunch friend, gentle in manner, full of consideration for others, a true and good physician.

Those who were associated with him alone understood how to value his unremitting and faithful service to his profession. He was just to all; his ideals were high.

His influence extended beyond the borders of this State. He was one of the strong men in the National body—the American Medical Association—in the National Academy of Medicine and in the Association of American Medical Colleges. In all of these bodies he sought at all times to elevate the standard of the medical profession.

In the medical school of which he was the honored dean during many years he labored unselfishly in behalf of the younger workers whom he stimulated to continue the work which he had so auspiciously commenced and so unselfishly continued.

This Central New York Medical Assocation has sustained a great loss in the death of Doctor Henry Darwin Didama; at this time it makes grateful acknowledgment of the great influence of Doctor Didama's character, ability and efforts in its behalf and that of the medical profession and expresses its grief at his departure.

RESOLVED, That a copy of these resolutions be sent to the family of the deceased, published in the official Journal of the Association and entered upon the minutes of our meeting.

HENRY L. ELSNER,
EDWARD B. ANGELL,
L. L. TOZIER,
COMMITTEE.

*Adopted at Buffalo, Oct.* 24, 1905.

EDWARD B. ANGELL, of Rochester, then read a paper entitled "The Cerebral Palsies of Childhood."

DISCUSSION.

DR. LOEB: As a general practitioner this paper appeals to me. The question of when to intervene in difficult labors is always a difficult question to decide. I call to my mind now one case of a very difficult, protracted labor. This child died within a very few hours of its birth, and from what I attributed at that time to some injury to the cerebellum, producing some motor paralysis. It had at the time of its birth a very peculiar respiration. It breathed in a gasping manner, and then would breathe rather regularly. and then would gasp again. In examining the child's head I found the caput was very markedly soft, and I always attributed the disturbance to some injury to the cerebellum.

C. E. LOW, of Pulaski: A few years ago I had a delivery that was somewhat difficult and slow and I used the forceps. We succeeded all right and there did not seem to be any difficulty, but during a subsequent visit my attention was called to the fact that the child could not use one of its arms, but under massage and electrical treatment it recovered the use of the arm. In a subsequent delivery I got the same result. I do not know whether I used forceps or not, but this second time there was the paralysis of the one arm. I did not see the case again, as the people moved out of the neighborhood. I understood that child recovered, but I do not think that this type of paralysis comes in the class with Dr. Angell's.

The secretary: Mr. President, the following named gentlemen have complied with the requirements of the constitution and are eligible to membership in the association: C. M. Briggs, Fairport; D. J. Corrigan, West Webster; J. P. DeLaney, Geneva; E. A. French, Rochester; Mark Heiman, Syracuse; C. P. Jennings, Macedon; A. M. Johnson, Rochester; C. E. Low, Pulaski; J. W. Magill, Rochester; G. W. McClellan, Canandaigua; N. D. McDowell, Rochester; W. W. Percy, Rochester; Charles Reitz, Rochester; W. D. Stanton, Webster; G. B. Stocker, Buffalo; L. A. Walker, Rochester; L. B. Darling, Palmyra.

Motion made and seconded that they be declared elected.

Carried.

The following papers were read by title:

Some Gastric Symptoms in Non-Gastric Diseases, Nathan W. Soble, Rochester.

Conservative Pelvic Surgery, Edmund C. Boddy, Rochester.

Pain in Gallstones, Joseph P. Creveling, Auburn.

The Non-Surgical Treatment of the Prostate Gland in Emergency Cases, J. Henry Dowd, Buffalo.

A Report of a Case of Foreign Body in the Bronchus, Francis E. Fronczak, Buffalo.

The Unequal Pupil, as an Early Sign of Paresis, Wm. C. Krauss, Buffalo.

E. B. Angell, Rochester: Before this meeting is adjourned I would particularly like to express my gratitude to the Erie County members for the very fine entertainment they have given us today. I started late and was successful enough to get in before the dinner was finished. We have had a very enjoyable time, and I move that the thanks of the Association be extended to the physicians of Erie County for the entertainment.

Motion seconded and carried.

Dr. Greenleaf: I move that the Association extend to the Buffalo Historical Society a vote of thanks for the courtesies extended to us to-day.

Dr. Elsner seconded the motion.

Carried unanimously.

The president: The Secretary will please transmit this to the Secretary or President of the Buffalo Historical Society.

John O. Roe, Rochester: I wish to express the pleasure of the society at the able manner in which our President has conducted the meeting.

The president: I think your thanks are due to Dr. Krauss and to some of the other members of the committee who have assisted him. Dr. Krauss has been untiring in his efforts, and I know you will all be glad to join me in thanking him for his efforts.

The Association then adjourned.

